# Improving Electronic Health Record Note Comprehension With NoteAid: Randomized Trial of Electronic Health Record Note Comprehension Interventions With Crowdsourced Workers

**DOI:** 10.2196/10793

**Published:** 2019-01-16

**Authors:** John P Lalor, Beverly Woolf, Hong Yu

**Affiliations:** 1 College of Information and Computer Sciences University of Massachusetts Amherst, MA United States; 2 Department of Computer Science University of Massachusetts Lowell Lowell, MA United States; 3 Department of Medicine University of Massachusetts Medical School Worcester, MA United States; 4 Bedford Veterans Affairs Medical Center Center for Healthcare Organization and Implementation Research Bedford, MA United States

**Keywords:** health literacy, crowdsourcing, natural language processing, information storage and retrieval, psychometrics, MedlinePlus

## Abstract

**Background:**

Patient portals are becoming more common, and with them, the ability of patients to access their personal electronic health records (EHRs). EHRs, in particular the free-text EHR notes, often contain medical jargon and terms that are difficult for laypersons to understand. There are many Web-based resources for learning more about particular diseases or conditions, including systems that directly link to lay definitions or educational materials for medical concepts.

**Objective:**

Our goal is to determine whether use of one such tool, NoteAid, leads to higher EHR note comprehension ability. We use a new EHR note comprehension assessment tool instead of patient self-reported scores.

**Methods:**

In this work, we compare a passive, self-service educational resource (MedlinePlus) with an active resource (NoteAid) where definitions are provided to the user for medical concepts that the system identifies. We use Amazon Mechanical Turk (AMT) to recruit individuals to complete ComprehENotes, a new test of EHR note comprehension.

**Results:**

Mean scores for individuals with access to NoteAid are significantly higher than the mean baseline scores, both for raw scores (*P=*.008) and estimated ability (*P*=.02).

**Conclusions:**

In our experiments, we show that the active intervention leads to significantly higher scores on the comprehension test as compared with a baseline group with no resources provided. In contrast, there is no significant difference between the group that was provided with the passive intervention and the baseline group. Finally, we analyze the demographics of the individuals who participated in our AMT task and show differences between groups that align with the current understanding of health literacy between populations. This is the first work to show improvements in comprehension using tools such as NoteAid as measured by an EHR note comprehension assessment tool as opposed to patient self-reported scores.

## Introduction

### Background and Significance

In recent years, many hospitals have adopted patient portals to make medical records available to patients. In particular, patient portals allow patients to access their electronic health records (EHRs). In a survey of studies related to patient access to their medical records, generally, patients who chose to see their records were satisfied with their contents [1-4] and felt greater autonomy about their care [1,5,6]. Granting patients access to their records also does not increase the workload of medical staff members [1,7-9]. Generally, patient access to EHRs can lead to positive health outcomes and greater understanding of their conditions [1,10,11]. However, EHRs and the progress notes that are included often contain complex medical jargon that is difficult for patients to comprehend. When given access to their notes, patients have questions about the meaning of medical terms and other concepts included in the notes [9,12]. Tools such as OpenNotes have promoted the inclusion of patient visit notes in patient portals, but simply including the notes may not be beneficial for patients if they have questions regarding the meaning of terms in the notes. Tools and resources that can define terms and provide lay definitions for medical concepts are needed as part of the move to make EHR notes available to patients so that they can understand the contents of their notes and their medical record.

Self-service educational materials are widely available, especially on the Web. There is a wealth of information related to medicine and health care on the internet, ranging from well-maintained ontologies with curated educational materials to Web-based discussion communities of patients that suffer from the same disease. With this information, patients with certain symptoms can find information about their condition on the internet. But is the wealth of information useful? That is, does simply having access to health information lead to better understanding? In this work, we test the usefulness of both passive and active interventions for assisting patients with understanding medical concepts. The passive system, MedlinePlus (MLP) [13], is a Web-based repository maintained by the US National Library of Medicine that includes information and definitions for clinical concepts, diseases, and other terms related to health care. MLP has been used in the past to promote patient education and provide patients with definitions and educational material to improve health literacy [14-17]. MLP is a large repository of high-quality health care information, but the user must search for the information that he or she is looking for. MLP does not automatically surface information for users.

NoteAid [18,19] is a freely available Web-based system developed by our team that automatically identifies medical concepts and displays their definitions to users. NoteAid has previously been shown to improve patients’ understanding of notes as measured by self-reporting [18,19].

In this work, our goal is to determine if access to NoteAid or MLP is associated with higher levels of EHR note comprehension. Do these interventions of educational materials improve a patient’s ability to comprehend his or her EHR note? In this work, we use the Amazon Mechanical Turk (AMT) microtask crowdsourcing platform to give AMT workers (Turkers) the ComprehENotes EHR note comprehension test [20], a set of questions designed to test EHR note comprehension. AMT is an increasingly popular tool for gathering research data [21-23] and recruiting participants for experiments, both in open-domain tasks [24,25] and medical-specific research [26-29]. Certain Turkers were not given 1 of the external resources, whereas others were provided with either MLP or NoteAid. Our results show that using NoteAid leads to significantly higher scores on the EHR comprehension test compared with the baseline population that was given no external resource. However, we found no significant difference between the Turkers with no resource and the Turkers who used MLP. Turkers were also asked to take the short Test of Functional Health Literacy in Adults (S-TOFHLA) to assess functional health literacy. All the Turkers scored *adequate health literacy*, the highest level for S-TOFHLA. This is the first work to quantitatively analyze the impact of tools such as NoteAid using a test of EHR note comprehension as opposed to self-reported scores.

In this work, we show that NoteAid has a significant impact on EHR note comprehension as measured by a test specific to that task. In addition, simply giving a patient access to sites such as MLP does not lead to significant improvements in test scores over a baseline group that had no external resources available to them. Finally, we analyze the demographics of the Turkers who completed our tasks. A regression model to predict test scores showed differences between demographic groups that align with the current knowledge regarding health literacy. For example, individuals that reported education of less than high school scored lower than average, whereas individuals that identified as white scored higher than average.

### Related Work

Health literacy is an important issue for patients. Low health literacy is a widespread problem, with only 12% of adults estimated to be proficient in health literacy [30]. The Institute of Medicine defines health literacy as “the degree to which individuals have the capacity to make appropriate decisions regarding their health” [31]. Patients with low health literacy often have difficulty with understanding instructions for medications from their doctors and have trouble navigating systems for making appointments, filling prescriptions, and fulfilling other health-related tasks [32,33]. In addition, having low health literacy has been linked to negative health outcomes in areas such as heart disease and fear of cancer progression [34,35].

It is important to be able to test a patient’s health literacy to identify those patients with low health literacy. Doctors can then provide these patients with educational materials to improve their understanding of medical terms and concepts. Testing health literacy is especially important with the proliferation of Web-based patient portals, where patients can access their EHRs and EHR notes directly. Giving a patient access to their EHRs and EHR notes without confirming that the patient can understand the content of the notes may lead to confusion and frustration with their health care experience.

There are a number of tests for health literacy, including the Test of Functional Health Literacy in Adults (TOFHLA) and the Newest Vital Sign (NVS) [36-38]. TOFHLA and its shortened form (S-TOFHLA) test comprehension and numeracy by providing scenarios to patients and constructing fill-in-the-blank questions by removing key terms from the scenario passages. NVS is a short test where patients are required to answer questions related to a nutrition label, to test whether the patient can navigate the label. These tests work well as screening instruments to identify patients who may have low health literacy, but they are broad tests and do not specifically test EHR note comprehension.

Although these and other tests are available, the only test that specifically targets a patient’s ability to comprehend their EHR notes is the ComprehENotes test [20]. The ComprehENotes test questions were developed using key concepts extracted from deidentified EHR notes. Questions were written by physicians and medical researchers using Sentence Verification Technique and validated using Item Response Theory (IRT) [39,40]. The test set is the first of its kind that specifically tests a patient’s ability to comprehend the type of content that is included in EHR notes.

## Methods

### Overview

**Figure 1:**
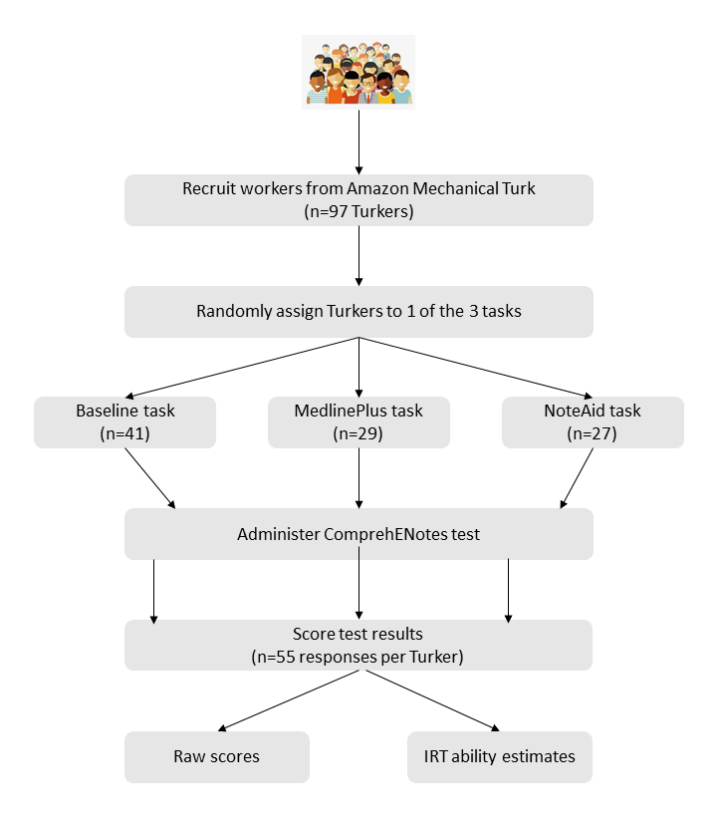
Flowchart describing our experiment. Amazon Mechanical Turk workers were randomly assigned to one of three tasks on the platform. They completed the ComprehENotes test with the use of the provided external tool. All scores were then collected, and ability estimated were obtained using Item Response Theory (IRT).

In this work, we recruited Turkers on the AMT platform and asked them to complete the ComprehENotes EHR note comprehension test. Turkers were split into 3 groups and were allowed to use 1 external resource when completing the test (or no resource in the case of the baseline group). Test results were collected and analyzed using IRT to estimate EHR note comprehension ability for each of the individuals, and group results were analyzed to determine if either of the external resources had a significant effect on test scores. Figure 1 illustrates our methodology at a high level. Details for each of the steps are described below.

### Data Collection

To assess EHR note comprehension, we used the ComprehENotes question set [20]. The dataset consists of 55 questions to measure EHR note comprehension. A bank of 154 questions was developed by groups of physicians and medical researchers from deidentified patient notes and then filtered down to a final test set using IRT. A total of 83 of the 154 questions were provided to AMT Turkers, who provided responses. These responses were used to fit an IRT model that estimated the questions’ ability to test EHR note comprehension. Of the questions in the original question bank, 55 were retained as a test of note comprehension [20].

The questions in the ComprehENotes test set include questions from patient EHR notes associated with 6 diseases: heart failure, hypertension, diabetes, chronic obstructive pulmonary disease (COPD), liver failure, and cancer. The questions are all general enough that they assess a key concept associated with 1 of the 6 diseases without being so specific to a single patient that they are not useful to others [20]. Therefore, the test can be used to assess a patient’s general EHR note comprehension ability and allows for comparisons between patients with respect to comprehension ability.

The ComprehENotes test set is most informative for individuals with low health literacy. That is, the SE of the ability estimation is lowest at low levels of ability (eg, −2 to −0.5). In addition, most of the ComprehENotes questions have low difficulty parameters. The difficulty parameters range from −2.2 to 0.7. That is, the questions are of a difficulty that individuals with lower than average ability have a 50% chance of answering correctly. For example, if a question has a difficulty parameter of −1.0, then an individual with estimated ability of −1.0 has a 50% chance of answering the question correctly. Ability estimates are normally distributed, so an individual with estimated ability of −1.0 is 1 SD below the average individual. Textbox 1 shows two example questions taken from the ComprehENotes test. Individuals are shown a snippet of text from a deidentified EHR note and asked to select the answer that has the same meaning as the italicized portion of the text.

We set up 3 AMT tasks for Turkers to complete. Turkers were presented with the ComprehENotes question set, 1 question at a time, and were asked to provide the correct answer.

**Figure 2:**
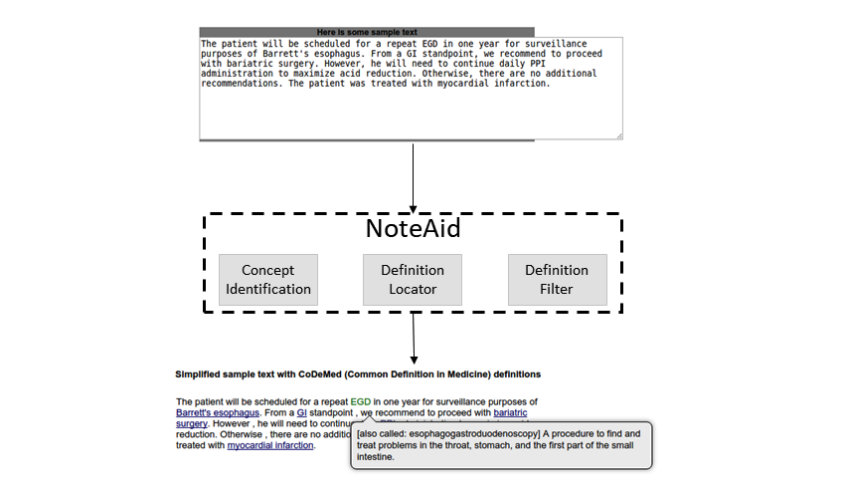
Example showing NoteAid simplified text.

For 1 task (Baseline), the Turkers were instructed to not use any external resources when answering the questions. For the first treatment task (Treatment-MLP), Turkers were given a link to MLP and were told that they could use the site as a reference when completing the task. Turkers were encouraged to use the MLP page search functionality to search for definitions to unknown terms or concepts that appeared in the task. For the second treatment task (Treatment-NoteAid [Treatment-NA]), the Turkers were provided with a version of the ComprehENotes test set that had been preprocessed with NoteAid. We preprocessed the ComprehENotes question text using NoteAid, extracted the simplifications and definitions that were provided, and used the NoteAid output as the question text shown to Turkers in the Treatment-NA group (refer to Figure 2 for an example of text simplified by NoteAid). The tasks were restricted so that individuals who completed 1 were not eligible to complete the other 2. For all groups, we collected demographic information about the Turkers’ age, gender, ethnicity, level of education, and occupation. We also administered the S-TOFHLA test for each group to assess functional health literacy and to compare S-TOFHLA and ComprehENotes scores.

As we are not able to monitor the Turkers as they complete our tasks, we cannot know for sure that the baseline group did not use any external resources as instructed. However, we can be confident that they did not have access to NoteAid. To access NoteAid, the Turkers would have to have known the URL link to access the system, even though we did not provide it to them. Alternatively, the Turkers would have had to search for NoteAid without knowing the name of the specific system we are testing. Therefore, we are confident that even if the baseline group did use some external source during the task, they did not have access to NoteAid. The baseline Turkers may have found MLP if they searched on the Web for medical concepts during the task. For example, a Google search of “COPD definition” returns an MLP link on the first page. However, unless the Turkers knew about MLP before beginning the task, it is unlikely that they would use MLP as a reference during the task.

**Sample questions taken from the ComprehENotes test.**

Instructions

Please read the following questions, making a note of the italicized text, and then examine the provided answer choices. Please select the answer that best represents the italicized portion of the question text.

Amitriptyline 25 mg po at bedtime; Bactrim 160 mg po bid on Friday, Saturday, and Sunday; hydrocortisone cream; and *pegfilgrastim 6 mg subcutaneous one dose*. He will continue to return for scheduled chemotherapy and will also be following up with the hematology and oncology clinic.

1. Do a under skin injection of 1 dose of 6 mg pegfilgrastim.
2. Pegfilgrastim 6 mg epidermal 1 dose.
3. Pegfilgrastim may prevent neutropenia.

*The patient is in for her physical examination today*. Overall, she is doing very well. She is not on any blood pressure medications at the moment; she is doing fine. She had some issues in the past, but those settled down. Her blood pressure is 110/78 today on no medications, pulse 68 and regular, respirations 12.

1. No physical examination was performed for the patient today.
2. The patient came to check her health.
3. An eye exam is not a part of a regular physical examination.

We included quality control checks for our AMT tasks to ensure a high-quality response from the Turkers. First, we restricted access to our tasks to Turkers with a prior approval rating above 95% to include only Turkers whose work has been judged as high quality by other requesters. We also restricted the task to Turkers located in the United States as a proxy for a test of English proficiency. Within the actual task, we included 3 quality-check questions, which consisted of a very simple question with an obvious answer. If any Turker answered 1 or more of the quality control checks incorrectly, their responses were removed from the later analyses.

### NoteAid

The NoteAid system supplies lay definitions for medical concepts in EHR notes [18,19]. Users enter the text from their EHR notes into the NoteAid system, which outputs a version of the note with medical concepts defined. When the user hovers his or her mouse over a concept, a popup with the definition is shown. Figure 2 shows a high-level overview of the components in the NoteAid system, with example text that has been annotated. Users enter their EHR note text into NoteAid and are provided with a reproduction of the text, with key medical concepts linked to their definitions.

**Figure 3:**
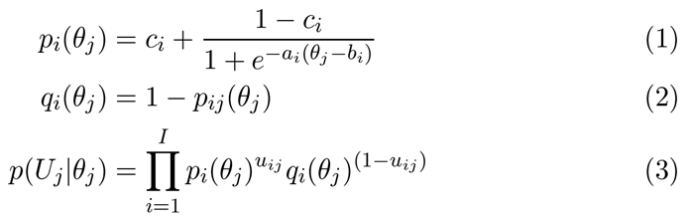
Equations for Item Response Theory 3-parameter logistic models.

NoteAid consists of 2 components. The *concept identifier* component processes input text and maps terms to medical concepts. The concepts are mapped to entries in the Unified Medical Language System using MetaMap [41,42]. It then filters the list of returned concepts to include only concepts that match a subset of possible semantic types related to patient health (eg, disease or syndrome and lab or test result). The *definition fetcher* component uses the filtered list of concepts to pull definitions from an external knowledge resource (eg, Wikipedia or MLP).

Previous evaluation of NoteAid has shown that patients’ self-reported comprehension scores improve when using the system [18,19]. However, there has not yet been an evaluation of NoteAid on a test of comprehension, as opposed to self-reporting scores.

### Item Response Theory Analysis

The ComprehENotes test set was developed using IRT [40]. The test set was built according to a single factor, 3-parameter logistic IRT model with a fixed guessing parameter. The test, therefore, measures a single latent trait, specifically the ability to comprehend EHR notes. Once the model has been fit, ability for a new test respondent is estimated by estimating *θ* according to the respondent’s answers to the test questions after the responses have been converted to a correct or incorrect binary format. For a single test question *i*, the probability that individual *j* answers the question correctly is a function of the individual’s ability (*θ*). Figure 3 includes 3 equations: equation 1 is used to calculate the probability that individual *j* with an estimated ability of *θ*_*j*_ will answer question *i* correctly; equation 2 calculates the probability that individual *j* with estimated ability *θ*_*j*_ will answer question *i* incorrectly; and equation 3 calculates the likelihood of individual *j* ’s set of responses *U*_*j*_ to all items in the test set, where *u*_*ij*_ is 1 if individual *j* answered item *I* correctly and 0 if they did not.

*p*_*i*_ and *q*_*i*_ are functions of the known item parameters, and therefore, we can estimate *θ* via maximum likelihood for each Turker. We also calculated raw test scores for each Turker (percent of questions answered correctly) for comparison.

## Results

### Turker Demographics

We first report the demographic information for the Turkers who completed our tasks. Table 1 shows the demographic information that we collected from the Turkers for the Baseline, Treatment-MLP, and Treatment-NA groups. Overall, most of the Turkers who completed our tasks are white, young, and have at least an associate degree. In addition, most of the Turkers do not work in the medical field. These demographics are not representative of a wider population and do not fit demographics that are more commonly associated with low health literacy [31]. However, our goal here is to compare the results with respect to different interventions. In this case, we do not need to test individuals with low health literacy; we instead want to see if scores improve when users are provided with certain external resources.

### Influence of Interventions

Our analysis includes both the raw test scores as well as the estimated ability level using IRT. As the test set consists of questions that were fit using IRT, we can also calculate the ability of these Turkers and test whether the mean ability score was higher for Turkers that used NoteAid. Ability is a useful metric as it takes into consideration which questions you answer correctly, not just how many. IRT models question difficulty, so by considering whether easy or difficult answers were correct, IRT allows for a more informative score than percent correct. For each Turker, we calculated their ability score (*θ*) using the IRT model fit as part of the ComprehENotes dataset [20]. We use the *mirt* and *ltm* open-source R packages for estimation [43,44].

**Figure 4:**
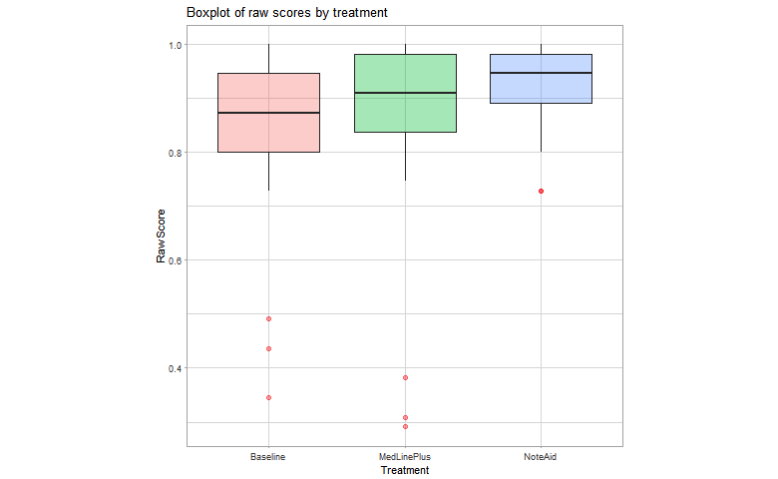
Box plot of raw scores for baseline and treatment Turker groups. The treatment groups were able to use MedlinePlus and NoteAid, respectively, when taking the ComprehENotes test.

Figure 4 plots the raw scores for each AMT Turker for our test set. The center rectangles span the range from the first quartile to the third quartile of responses, and the bolded line inside each box represents the median score. Open circles indicate outlier scores. The upper horizontal line marks the maximum score for each group, and the lower horizontal line is 1.5 times the interquartile range below the first quartile. As the figure shows, visually there is a spread between the populations that did and did not have access to the interventions. Median raw scores for the baseline and MLP groups are similar, whereas median scores for the NoteAid group is higher. The spread of responses for the treatment groups is also smaller than the baseline group.

**Figure 5:**
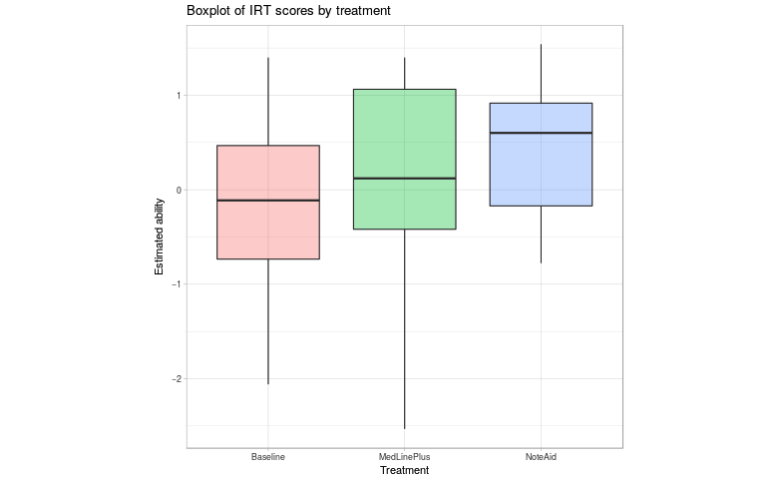
Box plot of ability estimates for baseline and treatment Turker groups. The treatment groups MLP and NA were able to use MedlinePlus and NoteAid, respectively, when taking the ComprehENotes test. IRT: Item Response Theory.

Figure 5 shows the box plots of ability estimates. Again, the median values for the baseline and MLP groups are similar and the median ability estimates for the NoteAid group is higher. The lowest ability estimates for the baseline and MLP groups are much lower than for the NoteAid group (2 SDs below the mean as opposed to 1 SD below). This shows that even for individuals that use NoteAid and still struggle, the low range of ability is higher than when NoteAid is not used.

To test whether either intervention caused a significant difference in scores, we compared each intervention with our baseline using Welch 2-sample *t* test. Table 2 shows the mean raw scores and mean ability estimates for Turkers in each group. Mean scores are significantly higher than the baseline for Turkers that had access to NoteAid, both with regard to the raw scores (*P*=.01) and estimated ability (*P*=.02).

### Regression Analysis

We also wanted to determine if demographic factors had an impact on test scores. To that end, we fit a linear regression model to predict raw scores using demographic information and group (eg, baseline or treatment) as features. The results of the analysis showed that the intervention (none, MLP, or NoteAid) was a significant feature in predicting raw score. In addition, certain demographic groups were significant in determining score. Regarding ethnicity, individuals who self-reported as white had a significant positive coefficient. Regarding education, individuals that have less than a high school degree had a significant negative coefficient. These results are consistent with what is known about populations that are at risk for low health literacy. Individuals with lower education often have higher instances of low health literacy, as well as minorities. Our populations for this task, particularly with regard to minorities and less educated individuals, were very small. Future work on NoteAid in minority populations would be worthwhile to confirm these effects.

**Table 1:** Demographic information collected from Turkers who completed our task.

Demographic		Baseline (N=41), n (%)	MedlinePlus (N=29), n (%)	NoteAid (N=27), n (%)	Total (N=97), n (%)
**Gender**					
	Male	27 (66)	8 (28)	18 (67)	53 (55)
	Female	14 (34)	21 (72)	9 (33)	44 (45)
**Age (years)**					
	22-34	23 (56)	16 (55)	16 (59)	55 (57)
	35-44	6 (15)	9 (31)	8 (30)	23 (24)
	45-54	8 (20)	2 (7)	3 (11)	13 (13)
	55-64	4 (10)	2 (7)	0 (0)	6 (6)
	65 and older	0 (0)	0 (0)	0 (0)	0 (0)
**Ethnicity**					
	American Indian or Alaska Native	0 (0)	1 (3)	1 (4)	2 (2)
	Asian	3 (7)	0 (0)	1 (4)	4 (4)
	Black or African American	8 (20)	3 (10)	4 (15)	15 (16)
	Hispanic	4 (10)	1 (3)	0 (0)	5 (5)
	White	26 (63)	24 (83)	21 (78)	71 (73)
**Education**					
	Less than high school	1 (2)	0 (0)	0 (0)	1 (1)
	High school diploma	9 (22)	8 (28)	8 (30)	25 (26)
	Associates	8 (20)	5 (17)	3 (11)	16 (17)
	Bachelors	20 (49)	14 (48)	14 (51)	48 (50)
	Masters or higher	3 (7)	2 (7)	2 (7)	7 (7)
**Occupation**					
	Physician	0 (0)	0 (0)	1 (4)	1 (1)
	Nurse	2 (5)	0 (0)	0 (0)	2 (2)
	Medical student	1 (2)	1 (3)	1 (4)	3 (3)
	Other profession in medicine	2 (5)	3 (10)	3 (11)	8 (8)
	Other profession	36 (88)	25 (86)	22 (82)	83 (86)

**Table 2:** Mean scores for the 3 groups. Mean NoteAid scores are significantly higher than the mean baseline scores, both for raw scores (*P*=.01) and estimated ability (*P*=.02).

Group	Raw score	Ability estimate
Baseline	0.831	−0.065
MedlinePlus	0.849	0.138
NoteAid	0.923^a^	0.477^a^

^a^Score significantly higher than baseline.

### Comparison With the Short Test of Functional Health Literacy in Adults

All Turkers who completed our tasks were also given the S-TOFHLA test to complete. Scores on S-TOFHLA place test-takers into 1 of the 3 categories: *inadequate health literacy*, *marginal health literacy*, and *adequate health literacy*. It is most useful as a screening tool to identify individuals with low or marginal health literacy. All Turkers in our tasks were scored to have *adequate health literacy*. In fact, all Turkers either scored perfect scores or only answered 1 question incorrectly, whereas the scores from the ComprehENotes test covered a wide range of ability estimates. The ComprehENotes can be used to assess EHR note comprehension at a more granular level as opposed to a screening tool such as S-TOFHLA, where the primary concern is identification of individuals with low health literacy.

### ComprehENotes Analysis

Finally, we wanted to see if the IRT model that was originally fit as part of the ComprehENotes dataset was validated by the response patterns that we collected from the Turkers. To this end, we selected the 2 questions that the most Turkers answered correctly as well as the 2 questions that the fewest Turkers answered correctly.

These questions can be considered the easiest and hardest, respectively, from our task. The difficulty parameters for these items as modeled by IRT match the expectation of how difficult these items should be. The 2 hardest questions from our task (in terms of how many Turkers answered correctly) have difficulty parameters of 0.7 and −0.3, whereas the 2 easiest questions have difficulty parameters of −1.8 and −1.4. The difficulty parameter is associated with the level of ability at which an individual has a 50% chance of answering the question correctly. Therefore, the low difficulty levels imply that someone of low ability has a 50% chance of answering the question correctly. Conversely, a higher difficulty parameter means that someone must be of a higher estimated ability level to have a 50% chance of answering correctly.

## Discussion

### Principal Findings

In this work, we have shown the importance of targeted, active intervention when trying to improve a person’s ability to comprehend EHR notes. By giving Turkers access to NoteAid, scores on the ComprehENotes test are significantly improved over a baseline population that had no external resources. On the other hand, Turkers that had access to MLP but had to search themselves for the information that they wanted did not have a significant improvement in scores. NoteAid automatically identifies key medical concepts and provides definitions, as opposed to the scenario with MLP, where a user must decide what to search for. The user may not know that a certain concept is key for understanding a passage or they may assume that they understand certain concepts that they do not. By letting the user decide what to search for, important terms may be missed and overall comprehension may be affected. This result is consistent with previous work on assessing comprehension using tools such as NoteAid [18,19], but this is the first time where the conclusion is based on an EHR note comprehension assessment instead of patient self-reported scores. By using the ComprehENotes test, we can quantitatively confirm the previous results self-reported by patients.

### Limitations

There are limitations to this work. First, by using AMT, we are not able to monitor the Turkers who complete our task to ensure that only the external resources that we provide were used. This is particularly true in the baseline group, where our expectation is that no external resource was used. However, it is unlikely that the baseline users were able to access NoteAid without prior knowledge of the system; therefore, we can be confident that they did not use it in our task. If the baseline users did use external resources, they most likely used a passive resource such as Google or even MLP. As NoteAid was integrated into the Treatment-NA task, we can be confident that Turkers in the Treatment-NA task used NoteAid. The discrepancy between Treatment-MLP and Treatment-NA may seem to bias improvements toward the Treatment-NA group, but there is an important distinction to be made. At present, sites such as MLP are available to any patient that seeks them out, but the onus is on the patient to go to the site and search for terms. With the Treatment-NA group, we have shown that by integrating a system that can simplify and define medical terms automatically, the burden of defining terms is removed from the patient.

In addition, the demographics of the Turkers who completed our task are not representative of the larger population, specifically among demographics associated with higher risks of low health literacy [31]. In the case of this work, that is not problematic, as our goal was to examine the effect of active and passive interventions on EHR note comprehension. The demographics of our 3 groups were similarly distributed, so the changes in scores can be linked to the intervention used. Although the results obtained were significant, ideally larger populations could be examined in each group. However, as the demographics of the Turkers are not consistent with demographic groups associated with low health literacy, the follow-up work should focus on those groups. By using AMT and Turkers, we have shown that tools such as NoteAid do improve EHR note comprehension generally, but future work should look specifically at groups associated with low health literacy to determine if our results hold for those groups as well.

Another limitation of this study is that patients are not evaluated on their own notes. Ideally, we would be able to assess the EHR note comprehension of each patient by testing the patient using concepts extracted from his or her own EHR notes. However, there are several roadblocks to making this a reality. First, this type of personalized assessment would reduce the ability to compare comprehension ability between patients. If a patient scores highly on an assessment of their own note, we can say that the patient understands the note, but if there were no complex concepts in the note, we cannot compare this with a patient who scores poorly on an evaluation based on his or her own complex EHR note. Second, to build a personalized EHR note evaluation would require complex natural language processing (NLP) systems to automatically generate multiple- choice questions (MCQs) for patients when they enter their EHR notes. To our knowledge, there does not currently exist an NLP system for medical MCQ generation. We do believe that the development of such a system will be beneficial for personalized patient assessment of EHR note comprehension. Such a personalized system could complement the ComprehENotes test so that a patient would be assessed on their own EHR note as well as on a standardized assessment.

### Conclusions

In this work, we have shown that simply having access to resources designed to improve health literacy and medical concept understanding is not enough to provide benefit. The Turkers in our experiment who had access to MLP did not score significantly higher on the ComprehENotes test than those Turkers that were not provided with an external resource. On the other hand, having access to NoteAid, which actively pulls definition information and provides it to the user, led to significantly higher scores for Turkers. This result validates previously reported self-scored comprehension results showing that users had an easier time understanding their notes when they had access to NoteAid.

Knowing that users do not see benefits from simply having access to MLP is an important observation. When doctors are recommending next steps for patients who wish to improve their health literacy, it may not be sufficient to point them to Web-based resources. Targeted interventions are necessary to ensure that patients are able to learn about specific concepts and diseases that are relevant to them. In particular, the integration of NoteAid with the EHR note on a patient’s portal would remove the friction from the patient accessing an external resource. Instead, the patient would have key terms defined and simplified within his or her own patient portal, which would minimize the effort involved from the patient’s standpoint and keep the information in the note within the portal itself.

There are several directions for future work. Developing target curricula is necessary to ensure that patients can see benefits from Web-based resources. They may not need a tool such as NoteAid (eg, if they are not looking at their notes), but something more targeted than MLP is needed to ensure that patients are learning. In addition, there should be further validation of the ComprehENotes test set with patients that are at risk for low health literacy. The Turkers in our task all scored either close to average or above average in our ability estimates, except for a few outliers. The test was designed to be most informative for individuals of lower ability, so this test should be replicated with such a population.

## Abbreviations

AMT: Amazon Mechanical Turk
COPD: chronic obstructive pulmonary disease
EHR: electronic health record
IRT: Item Response Theory
MCQ: multiple-choice question
MLP: MedlinePlus
NLP: natural language processing
NVS: Newest Vital Sign
S-TOFHLA: short Test of Functional Health Literacy in Adults
TOFHLA: Test of Functional Health Literacy in Adults
Treatment-NA: Treatment-NoteAid
